# Longitudinal analysis of *Plasmodium falciparum* genetic variation in Turbo, Colombia: implications for malaria control and elimination

**DOI:** 10.1186/s12936-015-0887-9

**Published:** 2015-09-22

**Authors:** Stella M. Chenet, Jesse E. Taylor, Silvia Blair, Lina Zuluaga, Ananias A. Escalante

**Affiliations:** School of Life Sciences, Arizona State University, Tempe, AZ USA; School of Mathematical and Statistical Sciences, Arizona State University, Tempe, AZ USA; Malaria Group, Universidad de Antioquia, Medellín, Colombia; Institute for Genomics and Evolutionary Medicine, Temple University, Philadelphia, PA USA

**Keywords:** Malaria incidence, *Plasmodium*, Mutation rate, Microsatellites, Inbreeding

## Abstract

**Background:**

Malaria programmes estimate changes in prevalence to evaluate their efficacy. In this study, parasite genetic data was used to explore how the demography of the parasite population can inform about the processes driving variation in prevalence. In particular, how changes in treatment and population movement have affected malaria prevalence in an area with seasonal malaria.

**Methods:**

Samples of *Plasmodium falciparum* collected over 8 years from a population in Turbo, Colombia were genotyped at nine microsatellite loci and three drug-resistance loci. These data were analysed using several population genetic methods to detect changes in parasite genetic diversity and population structure. In addition, a coalescent-based method was used to estimate substitution rates at the microsatellite loci.

**Results:**

The estimated mean microsatellite substitution rates varied between 5.35 × 10^−3^ and 3.77 × 10^−2^ substitutions/locus/month. Cluster analysis identified six distinct parasite clusters, five of which persisted for the full duration of the study. However, the frequencies of the clusters varied significantly between years, consistent with a small effective population size.

**Conclusions:**

Malaria control programmes can detect re-introductions and changes in transmission using rapidly evolving microsatellite loci. In this population, the steadily decreasing diversity and the relatively constant effective population size suggest that an increase in malaria prevalence from 2004 to 2007 was primarily driven by local rather than imported cases.

**Electronic supplementary material:**

The online version of this article (doi:10.1186/s12936-015-0887-9) contains supplementary material, which is available to authorized users.

## Background

During the 1970s and 1980s, malaria incidence increased in South America due to the disorganized development of cities and the emergence of drug resistance in *Plasmodium falciparum* [1, 2]. However, during the last 10 years, transmission has decreased and many areas exhibit limited spatial connectivity between parasite populations [3]. As a result of this tangible reduction in transmission, malaria control programmes in these areas are expected to change from the control to the elimination phase.

Previous studies of *P. falciparum* in South America identified strong genetic structure and epidemic expansions that were the result of inbreeding and bottlenecks due to high drug pressure [4–7]. As a result, parasite populations within this region exhibit high genetic differentiation. Regardless of these discoveries, there have been few attempts to incorporate information on parasite genetic diversity into control programmes [8–12]. A recent example is the study by Daniels et al. in Senegal [11]. This study illustrated how genetic information obtained by using SNPs on *P. falciparum* samples collected over an 8 years period enriched epidemiological information [11, 12]. In particular, the authors could detect genetic changes in the parasite population that correlated with field data by using epidemiological models [11].

In this paper, the relationship between parasite genetic variation and malaria epidemiology is explored in a locality that has seen substantial fluctuations in malaria prevalence. This study was conducted on samples collected over 8 years in the northwest region of Colombia, one of the most malaria-endemic countries in South America [13]. Due to economic activities, such as mining and agriculture, there is substantial migration of workers with very high exposure to malaria (in close proximity to *Anopheles sp.* breeding sites) into different towns. These local movements are considered important in the maintenance of malaria transmission in Colombia and other similar settings in the Americas [1, 14]. Samples were genotyped at several microsatellite loci and analysed using Bayesian coalescent methodologies that have been successfully applied to other human pathogens [15–17]. In contrast to cross-sectional studies, genetic data from longitudinal historical samples can be used to separately estimate the effective population size (*N*_*e*_) and substitution rate at each locus. In addition, other mutations associated with drug resistance in *P. falciparum* were explored to determine how their frequency changed after the drugs were no longer in use.

## Methods

### *Plasmodium* isolates

A total of 257 *P. falciparum* blood samples collected on filter paper between 2002 and 2009 from surveillance studies carried out in Turbo (Antioquia department, Colombia) were genotyped: 94 samples were collected between October 2002 and July 2003, 80 from January 2004 to December 2005, 47 between April and October 2007, and 36 between March 2008 and July 2009. All samples came from *P. falciparum*-positive patients diagnosed by blood smear that gave their consent to participate in therapeutic efficacy studies at their local hospital [14, 18]. The samples used were those collected prior treatment [14, 18]. The inclusion criteria for the participants were: (a) patients that were older than 1 year of age with fever that lasted 48 h, (b) the patients were living permanently in the study site, and (c) none had severe malaria or were pregnant. The exclusion criteria were: (a) having another associated disease requiring additional treatment, (b) having mixed *Plasmodium vivax* and *P. falciparum* infections, (c) being hypersensitive to anti-malarial drugs, (d) moving to another site different from that where the study was conducted, (e) voluntary withdrawal from the study, (f) and third-party administration of anti-malarial drugs [19]. All patients received treatments approved by the Colombian authorities for uncomplicated malaria at the time: amodiaquine (AQ) plus sulfadoxine-pyrimethamine (SP) until 2006, artesunate (AS) plus mefloquine (MQ) in 2007, and artemether plus lumefantrine (LF) (Coartem^®^) from 2008 forward.

### DNA isolation and genotyping methods

DNA was isolated using the QIAamp DNA mini kit (QIAGEN, Valencia, CA, USA) and whole genome amplification was performed using the Repli-g Mini Kit (QIAGEN, Valencia, CA, USA). Twelve neutral microsatellites distributed across seven chromosomes were used in this study [19]. Fluorescent-labelled PCR products were separated on an Applied Biosystems 3730 capillary sequencer and scored using Gene Marker v1.95 (SoftGenetics LLC). The discovery of more than one allele in a sample was interpreted as a multiclonal infection. Missing data (no amplifications) were reported by locus but not considered for defining haplotypes.

The samples were also genotyped by direct sequencing to evaluate mutations at *Pfcrt* (codons 72–76), *Pfdhfr* (codons 50, 51, 59, 108, and 164) and *Pfdhps* (codons 436, 437, 540, 581, and 613) which have been associated with chloroquine, pyrimethamine, and sulfadoxine resistance, respectively. Only samples from single infections as determined by neutral microsatellite loci were sequenced. The PCR primers and conditions for *Pfcrt*, *Pfdhfr* and *Pfdhps* have been previously described [20, 21]. In addition, all samples were assayed for 15 microsatellite loci that span 129 Kb on chromosome 4 around *Pfdhfr*, 15 loci that span 139 Kb on chromosome 9 around *Pfdhps* [22] and ten loci that span 500 Kb on chromosome 7 around *Pfcrt* [20].

### Population genetic analyses

Genetic variation at each locus was quantified by the allelic diversity, which was estimated using the formula ${\text{H}}_{\text{E}} = [{\text{n}}/({\text{n}} - 1)]\left[ {1 - \sum\nolimits_{i = 1}^{L} {p_{i}^{2} } } \right]$, where n is the number of isolates sampled and p_i_ is the frequency of the ith allele (i = 1, …, L) in the sample. The sampling variance for H_E_ was calculated as $2(n - 1)/n^{3} [2(n - 2)]\left[ {\sum\nolimits_{i = 1}^{L} {p_{i}^{3} } - \left( {\sum\nolimits_{i = 1}^{L} {p_{i}^{2} } } \right)^{2} } \right]$ [23]. To assess the parasite population structure, the genetic data was analysed with Structure v2.1 [24], which uses a Bayesian clustering algorithm to assign isolates to a fixed number (*K*) of populations or clusters. The data were evaluated using different *K* values (*K* = 2–10) and ten independent chains were run for each *K* value with a burn-in period of 10,000 iterations followed by 50,000 iterations. The admixture model was used in all cases, allowing for the presence of individuals with ancestry in two or more of the K populations. The population was also described using median-joining networks inferred by Network 4.6.1.1 [25] on neutral and linked microsatellite data. The standardized index of association (${\text{I}}_{\text{A}}^{\text{S}}$) was also used to test for evidence of overall multi-locus linkage disequilibrium in the population per year [26]. FST values were calculated using Arlequin 3.11 [27].

### Bayesian phylogenetic analyses: microsatellite substitution rates and phylodynamics

Microsatellites can gain and lose repeats by two different mechanisms: unequal crossing over and DNA-replication slippage [28]. Bayesian coalescent-based method implemented in BEAST v.1.7.5 [29] were used to compare 12 models of microsatellite evolution, which make different assumptions about the relationship between the expansion and contraction rates and the repeat length [30]. In addition, the mean substitution rate at each locus in units of absolute time was estimated. The tip dates (sampling dates) were recorded in months and the coalescent constant population size model was used with a strict molecular clock and a standard uniform prior on the mean substitution rate. Once all 12 microsatellite mutation models had been run in this fashion, the model best supported by the data was identified by calculating the Bayes’ factor for each model relative to a reference model and choosing the one with the largest factor.

The best supported substitution model and the corresponding substitution rate were used in an Extended Bayesian Skyline Plot (EBSP) to estimate the parasite effective population size (*N*_*e*_) through time, assuming a generation time for malaria infections of a month. The EBSP assumes a non-parametric piecewise linear model of population size and uses information from multiple unlinked neutral loci to estimate the number of population size changes. Each analysis was performed with three independent runs based on 8 × 10^8^ generations and samples taken every 1 × 10^6^ steps after a burn-in period of 20 %. Convergence of the Markov chains was assessed by monitoring both the trace plots and effective sample sizes.

## Results

### Genotyping of neutral microsatellite loci

A total of 245 out of 257 samples amplified at 11 out of the 12 loci. Nine loci were retained for further analysis due to their clear pattern on the electropherogram (single peak) that facilitated the identification of alleles (Additional file 1). Of these, five exhibited high levels of variation (Additional file 1). Although 57 multiple infections were detected, 90 % of these were variable at only one locus suggesting that these patients were infected by highly related parasites (Table 1). A total of 79 different multi-locus genotypes were identified and analysed (Additional file 2), those included four genotypes that were found in at least six consecutive years following 2003 (Additional file 2). However, most pairwise fixation indices calculated between samples collected in different years were significantly different from zero (Table 2), indicating substantial changes in the genetic composition of the parasite population between years. Genetic diversity (H_E_ = 0.35 ± 0.11, Table 1) was highest in samples collected in 2002, with 93.3 % of infections caused by different lineages, after which H_E_ steadily declined in all years except 2008. Several unique haplotypes were found in low frequency each year (Additional file 3). Finally, analysis of the microsatellite data using the index of association statistic detected significant multilocus linkage disequilibrium (LD) in every year (${\text{I}}_{\text{A2003}}^{\text{S}}$ = 0.058, p < 0.01; ${\text{I}}_{\text{A2004}}^{\text{S}}$ = 0.056, p < 0.01; ${\text{I}}_{\text{A2005}}^{\text{S}}$ = 0.14, p < 0.01, ${\text{I}}_{\text{A2007}}^{\text{S}}$ = 0.072, p < 0.01; ${\text{I}}_{\text{A2008}}^{\text{S}}$ = 0.143, p < 0.01; ${\text{I}}_{\text{A2009}}^{\text{S}}$ = 0.163, p < 0.01) except 2002, possibly because of the small number of samples collected that year. Significant genetic differences between samples per year are shown in Table 2.

**Table 1:** *Plasmodium falciparum* genetic diversity per year

Year	Number of cases	Sample size	Multiple infections^a^ (%)	Haplotypes^b^ (%)	Unique haps^c^	He	SD	Number of alleles	SD
2002	864	15	3 (20)	14 (93.3)	9	0.35	0.11	2.44	1.24
2003	406	79	19 (24.1)	32 (40.5)	17	0.31	0.09	3	1.12
2004	365	40	11 (27.5)	23 (57.5)	11	0.29	0.1	2.44	1.74
2005	349	31	6 (19.4)	16 (51.6)	6	0.28	0.09	2.11	1.62
2007	856	46	10 (21.7)	15 (32.6)	8	0.22	0.08	3	1.41
2008	220	20	3 (15)	9 (45)	4	0.25	0.07	2.44	0.88
2009	154	14	5 (35.7)	5 (35.7)	0	0.18	0.07	2.33	0.71

^a^Total number of multiple infections with lineages that differed at one or more loci

^b^Number (percentage) of different haplotypes in the total number of samples

^c^Unique haplotypes found per year

**Table 2:** Microsatellite-based genetic differentiation (Fst) between years

Year	2002	2003	2004	2005	2007	2008
2003	0.08*					
2004	0.20*	0.07*				
2005	0.19*	0.11*	0.11*			
2007	0.27*	0.18*	0.19*	0.27*		
2008	0.18*	0.08*	0.09*	0.19*	0.08*	
2009	0.29*	0.15*	0.15*	0.29*	0.02	0.02

*P value <0.05

### Genotyping of *Pfcrt, Pfdhfr* and *Pfdhps*

Most of the samples were double mutants (75E and 76T) for *Pfcrt*. There was also one triple mutant (74I, 75E and 76T) and one sensitive (wild type) genotype in the 2007 samples. For *Pfdhfr*, pyrimethamine resistance-associated mutations N51I and S108N were found in all samples except in one, also from the 2007 group. For *Pfdhps*, only sensitive and single mutant A437G genotypes were found. These drug-resistant genotypes appeared to be conserved over time (around 85 % of the samples from each year had the *Pfdhps* S**G**KAA genotype) regardless of the turnover of lineages revealed by neutral microsatellites. The allelic variation in the microsatellite loci flanking these resistance genes was also explored and found 29 different haplotypes for *Pfcrt,* 28 for *Pfdhfr* and 29 for *Pfdhps* (Additional files 4, 5, 6).

### Cluster analysis

**Fig. 1:**
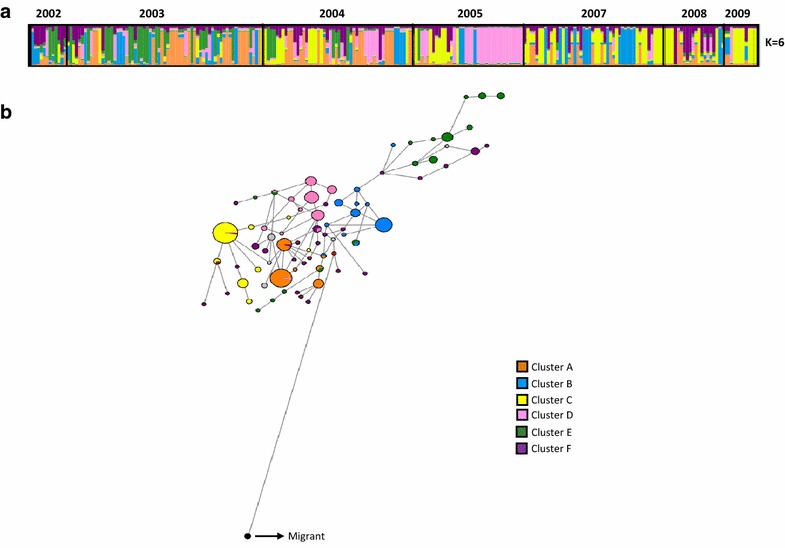
Population structure using neutral microsatellite loci. **a** Clustering per year using Structure 2.3. *Each colour* represents a different population cluster. **b** Median joining haplotype network. The haplotypes are represented by *circles* with width being proportional to their frequencies. The links are character differences and the *red circles* represent median vectors required to connect sequences within the network with maximum parsimony

Six clusters were identified, most of which persisted for several years (Fig. 1a; Table 3). While all six clusters were found in samples from 2002 to 2003, only five were detected in samples from 2007 to 2009, and a clear expansion of cluster D was observed in 2005. In contrast, cluster E was not detected in 2007, 2008 or 2009, and may have suffered a random local extinction during a period when the parasite population size was reduced following the introduction of AQ.

**Table 3:** Microsatellite-based genetic differentiation (Fst) between clusters

	A	B	C	D	E
B	0.08*				
C	0.05*	0.11*			
D	0.18*	0.15*	0.31*		
E	0.08*	0.09*	0.17*	0.12*	
F	0.02*	0.07*	0.00	0.24*	0.10*

*P value <0.05

**Fig. 2:**
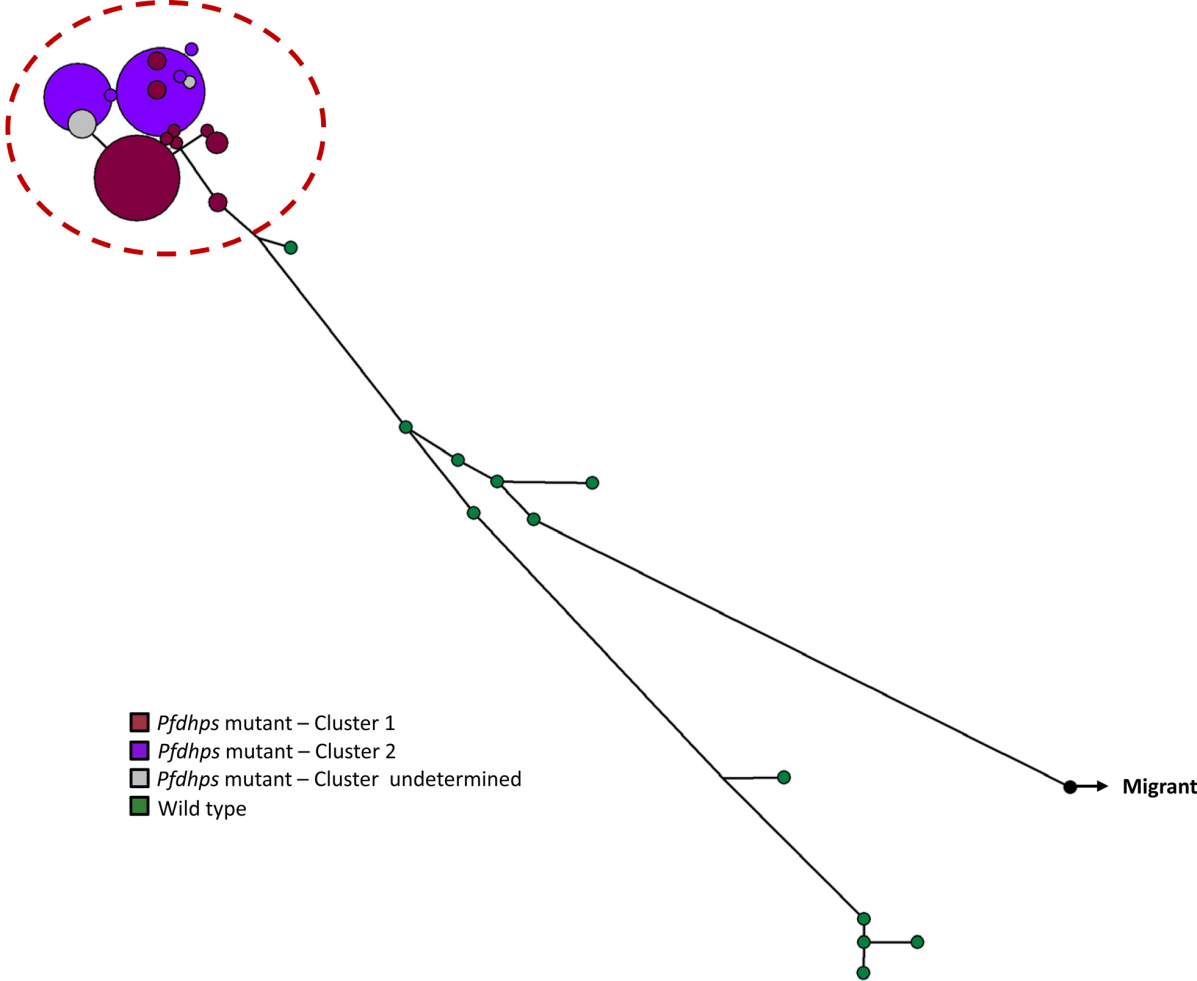
Median joining haplotype network using *Pfdhps*-linked microsatellites. The haplotypes are represented by *circles* with width being proportional to their frequencies. Haplotypes in the *red dashed circle* are single mutants grouped in *colour* clusters according to their genotypes (using Structure). Haplotypes in* green* are wild type genotypes

The software Network version 4.6.1.1 was used to characterize the relationship between multilocus genotypes (Fig. 1b) and those clusters that persisted for several years. Genotypes assigned to cluster F were highly dispersed in the network without a clear grouping pattern. One sample was distantly related to the rest of the network and had a sensitive genotype for *Pfcrt*, *Pfdhps* and *Pfdhfr*, which is present in Central America. Additionally, haplotype networks were constructed using the microsatellite loci flanking the drug resistance genes. The haplotypes associated with the *Pfdhps* mutant were grouped together, while the haplotypes associated with the wild type *Pfdhps* genotype were highly dispersed throughout the network (Fig. 2). This pattern was not observed in *Pfcrt* and *Pfdhfr* where the resistant genotypes were fixed.

### Time estimation and mutation rates

A total of 12 different microsatellite mutation models implemented in BEAST v1.7.5 were tested and the PL1 was the best supported model at each of the nine neutral loci included in this study (Table 4). Under this model, the substitution rate is linearly proportional to microsatellite length (P), the probability of microsatellite contraction is different from expansion but dependent on allele length (L), and each mutation changes the length of the microsatellite by exactly one repeat (1). Consequently, PL1 was used in all subsequent analyses conducted using BEAST. The mean substitution rate was estimated at each of these loci, which varied between 5.35 × 10^−3^ and 3.77 × 10^−2^ substitutions/month for the six most variable loci (mean of the posterior distribution; Table 5). Surprisingly, somewhat higher rates were estimated at three loci that harbored little variation, but the 95 % highest probability density (HPD) intervals were also much broader at these loci than at the other six, suggesting low information content in the data and greater influence of the prior distribution on the substitution rate.

**Table 4:** Models of microsatellite evolution using neutral microsatellite loci

Mutation model^a^	ln P (model|data)	SE	log_10_ (Bayes factor)^b^
EU1	−293.01	±1.986	11.330
EU2	−302.502	±2.594	15.420
EC1	−270.358	±2.919	1.492
EC2	−300.767	±1.168	14.698
EL1	−289.253	±1.263	9.698
EL2	−322.453	±2.943	24.116
PU1	−303.904	±2.57	16.000
PU2	−315.611	±3.152	21.145
PC2	−802.399	±67.545	9.644
PL1	−266.923	±0.732	0.000
PL2	−328.27	±4.27	26.643

^a^PC1 model was not included due to lack of convergence

^b^Bayes factors were computed relative to model PL1 using the formula: B.F.(X) = P(model PL1|data)/P(model X|data)

**Table 5:** Average microsatellite substitution rates (events/month) estimated using the PL1 model implemented in BEAST

Loci	POLYa	TA60	ARA2	Pfg377	PfPK2	TA109	TA81	TA42	2490
Global He	0.57	0.0078	0.58	0	0.65	0.0078	0.48	0.03	0.52
Mean	3.77E−02	0.1393	1.06E−02	0.2046	3.25E−02	0.4292	5.35E−03	8.00E−03	2.18E−02
Std err of mean	1.11E−03	4.75E−03	3.89E−04	7.57E−03	7.09E−04	2.19E−02	1.26E−04	3.77E−04	5.91E−04
Median	3.40E−02	8.97E−02	9.57E−03	0.1424	3.00E−02	0.3233	4.72E−03	6.39E−03	1.99E−02
95 % HPD lower	9.01E−03	6.40E−04	1.78E−03	2.86E−03	1.04E−02	5.20E−05	8.26E−04	1.42E−04	4.68E−03
95 % HPD upper	7.37E−02	0.4273	2.13E−02	0.5831	6.09E−02	1.1515	1.16E−02	2.07E−02	4.29E−02
Effective sample size (ESS)	275.038	1230.751	202.309	794.978	393.838	322.162	595.85	286.73	335.739

The migrant haplotype was excluded from calculations

**Fig. 3:**
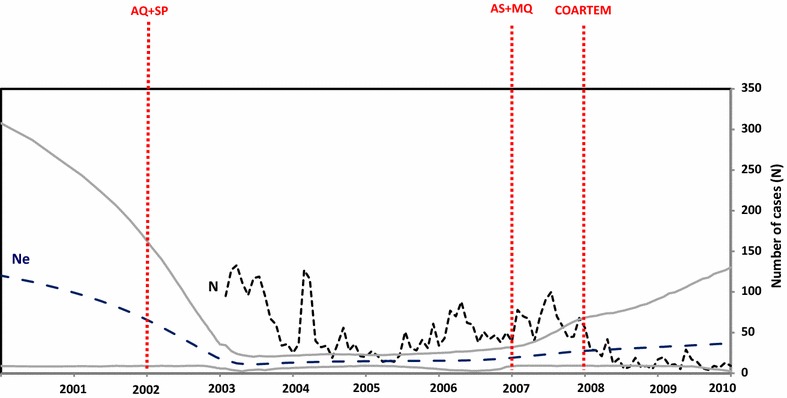
Comparison of the effective population size for *Plasmodium falciparum* with the number of reported cases in Turbo. The parasite generation time is assumed to be equal to a month. The mean and the 95 % HPD of the effective population size (*Ne*) estimated by the EBSP are represented by the *blue* and enclosing *grey lines*, respectively. The monthly number of *P. falciparum* cases in Turbo is represented by a *dashed black line* with changes in treatment regimen indicated by *red dashed vertical lines* (amodiaquine (AQ) plus sulfadoxine–pyrimethamine (SP), artesunate (AS) plus mefloquine (MQ), and artemether plus lumefantrine (Coartem^®^)

Examination of the extended Bayesian skyline plot (EBSP; Fig. 3) reveals that there is little correspondence between the estimated *N*_*e*_ (assuming a generation time of a month) and the number of monthly cases reported from 2002 to 2009 to the Antioquia Sectional Health Directorate (*Dirección Seccional de Salud de Antioquia*), Colombia. Neither the short-term fluctuations nor the longer-term trends seen in the monthly case data are evident in the EBSP. Furthermore, while the estimated *N*_*e*_ is several times smaller than the number of monthly case reports in most months between January 2002 and January 2007, the opposite pattern is seen in the period 2007 to 2009 when there was a pronounced decrease in the number of reported cases but also slight increase in the estimated effective population size.

## Discussion

The prevalence of *P. falciparum* malaria in Turbo, Colombia fluctuated dramatically between 2003 and 2010 (Fig. 3). These demographic changes were contemporaneous with changes in treatment protocols, which saw the adoption of new drug therapies in 2002, 2007 and 2008. However, as can be seen in Table 1a, the relationship between the treatment protocol and annual malaria prevalence is not simple. For example, whereas the switch to AS + MQ in 2007 had little immediate impact on the number of reported cases, the switch to COARTEM in 2008 coincided with a sharp decline in the number of cases, which decreased from 856 in 2007 to 220 in 2008.

To better understand the processes influencing *P. falciparum* malaria in this region, this study involved the characterization of this parasite population using a combination of neutral microsatellite markers and loci linked to known drug resistance mutations at three loci. Because these two classes of loci are expected to differ in both their mutation rates and exposure to selection, they have the potential to reveal different features of malaria epidemiology.

The analysis of the microsatellite data revealed the existence of several related multilocus genotypes that persisted for at least 6 years in the region around Turbo (Fig. 1; Additional file 2). This is the pattern expected in a parasite population where control efforts have left behind lineages that became the founders of clusters of highly related parasites [7, 10]. This observation differs from a previous study of a *Plasmodium* population in Peru which found that multilocus genotypes were rapidly broken down by recombination even in low-transmission areas [31]. Furthermore, all of the multilocus clusters detected in the final year of this study (2009) were also represented in 2002, suggesting that there was little immigration of novel parasite genotypes from outside of the region.

In low-transmission areas, meiotic recombination is most likely to occur between lineages that are closely related, leading to high levels of inbreeding. This is consistent with the fact that many of the putatively multiple infections identified in our sample vary at a single locus, possibly due to mitotic mutations that have occurred within the infected individual. Such mitotic events can lead to an overestimation of the number of observed multiple infections [8]. Inbreeding can also account for the significant levels of multilocus linkage disequilibrium documented in every year except 2002 (see “Results”). On the other hand, the microsatellite data suggests that there has been some recombination among the different clusters segregating in Turbo, as is evidenced by the admixture patterns seen in the cluster analysis (Fig. 1).

Although several multilocus haplotypes were observed to persist for multiple years, the cluster analysis revealed pronounced changes in the genetic composition of the *P. falciparum* population in Turbo from year to year, with different clusters dominant in different years. This observation is confirmed by the pairwise fixation indices shown in Table 2, which show significant genetic differentiation between every pair of years except between 2009 (when only 14 individuals were sampled) and the preceding 2 years. Rapid non-directional changes in the genetic composition of a population, such as observed here, can be explained by demographic stochasticity (i.e., genetic drift), which is expected to be pronounced in small populations. This is consistent with the low effective population size revealed by the EBSP (Fig. 3).

There is a lack of a correspondence between the estimated *Ne* displayed by the EBSP (using the posterior median of the EBSP) and the monthly case reports. During 2003 to 2007, *N*_*e*_ remained relatively unchanged even though there were substantial fluctuations in prevalence. Furthermore, between 2007 and 2009 there was a gradual rise in *N*_*e*_ even though the number of monthly case reports rapidly decreased following 2008. The reduction in cases coincides with an intense malaria control programme known as “*Papa Luis*” carried out in Antioquia between 2007 and 2009. This programme consisted of massive administration of chloroquine (an effective drug to treat *P. vivax* but not *P. falciparum*), biological control of *Anopheles* larvae, swamp drainage and indoor residual spraying [32]. During this period, the introduction of artemisinin-based combination therapy (ACT) is also thought to have contributed to a reduction in malaria cases.

The discrepancy between the epidemiological and genetic estimates could be explained by several factors. For parasites, *N*_*e*_ depends not only on prevalence, but also on the transmission rate and the variance in the number of new cases transmitted by infected individuals [9, 10, 33, 34]. In particular, a fixed parasite generation time of 1 month was assumed for the study, but the time between transmission events could vary affecting the parasite generation time. Similarly, the number of new cases generated by infected individuals can vary over time due to changes in social dynamics or public health policy and this too will affect *N*_*e*_. The observation that during 2003 to 2007, *N*_*e*_ remained relatively unchanged even though there were substantial fluctuations in prevalence suggests that seasonal clonal expansions led to a high prevalence at some time points. Indeed, frequent clonal expansions could even reduce *N*_*e*_ if they were accompanied by an increase in either the transmission rate or the transmission variance between infected individuals.

Another factor that may have contributed to the mismatch between the estimated values of Ne and the case report numbers is the variation in the sample sizes collected for genetic analysis in different years. In particular, the apparent rise in the estimated *N*_*e*_ from 2007 to 2009 may be due to the smaller number of samples collected in these years compared with the period 2003 to 2006. One consequence of the variable sample sizes is that the posterior distribution of the effective population size is more strongly influenced by the prior distribution in those periods when the data are less informative. Indeed, this is evident in the increasing credibility intervals for *N*_*e*_ between 2007 and 2009. Because of this sensitivity to sample sizes, archiving samples for genotyping should be a standard policy since those will facilitate understanding potential re-introductions or changes in prevalence.

The genetic data from samples collected over a span of 8 years also allowed to estimate microsatellite substitution rates separately from *N*_*e*_. At the six polymorphic loci with enough information, estimates ranged from 5.35 × 10^−3^ to 3.77 × 10^−2^ substitutions per locus per month (Table 5). In contrast, Su et al. estimated a genome-wide average microsatellite mutation rate of 1.59 × 10^−4^ per locus per meiosis from a genetic cross that was typed at 901 loci [35]. Notably, this rate falls below the 95 % HPD interval of five of the six loci that were investigated. The large discrepancy between these estimates could be due to two differences between the studies. First, whereas Su et al. only considered changes arising during meiosis, the estimates reported here reflect the accumulation of mutations over month-long periods during which the parasite is likely to have undergone multiple mitoses and at most one or two meioses. For the purposes of demographic/epidemiologic inferences, this composite rate is more relevant than the meiotic mutation rate. Secondly, locus-specific variation in microsatellite substitution rates could also explain this discrepancy. Since variable loci were chosen, those may be evolving more rapidly than the genome-wide pool of loci analysed by Su et al.

It should be noted that the mean substitution rates reported in this paper were obtained using the model best supported by our data, the single-phase proportional linear model (PL1). There was no support for any of the two-phase models of microsatellite evolution implemented in BEAST in which alleles may expand or contract by more than one repeat in a single step, although this too could be specific to the loci analysed in this study. The PL1 model assumes that the overall substitution rate is proportional to the repeat length, that each mutation results in the gain or loss of a single repeat, and that the probability of an expansion is a decreasing linear function of the repeat length, so that above a certain threshold, mutations are more likely to result in a contraction rather than in an expansion of the microsatellite [30]. As such, the rates reported in Table 5 are estimates of the mean substitution rate with respect to the stationary distribution of the microsatellite length under this model. These estimates provide empirical evidence that polymorphic microsatellite loci can be used to make inferences at time scales that are epidemiologically relevant [36].

Lastly, although drug resistance mutations can decrease in frequency once the corresponding drugs are no longer in use [37, 38], there are also endemic areas where such mutations have gone to fixation from intermediate frequencies even after drug pressure has been removed [4, 5, 20]. This study provides evidence of this process. Moreover, the data show that drug resistance mutations were fixed in a parasite population with a low effective population size and then remained at high frequency even after the drug policy was changed. Thus, anti-malarial drugs will remain ineffective unless there is an influx of sensitive parasites from other areas (e.g., Central America). The finding of a sensitive and genetically unrelated parasite demonstrates that such re-introductions are possible. It is worth noting that sensitive *Pfdhps* genotypes were still segregating in the population.

## Conclusion

This study provided an opportunity to observe changes in the genetic composition of a local *Plasmodium* population over many parasite generations. Several multilocus genotypes were found to be stable for many years, a dynamic that should be considered when genotyping is used to separate local from re-introduced cases in areas with seasonal or low malaria transmission. The estimated substitution rates reported in this study support previous claims that polymorphic microsatellite loci evolve on a timescale that facilitates epidemiologic investigations. Regardless of spikes in prevalence followed by the scale-up of interventions and/or changes in drug policy, the parasite effective population size remained small and relatively unchanged for many years. This suggests that the observed fluctuations in the number of cases were likely the result of local processes (e.g. operational or environmental factors) that affected malaria transmission. Such processes will be better understood by incorporating genetic data into epidemiological models [11]. Furthermore, mutations associated with drug resistance remained fixed in the population demonstrating that, without the influx of sensitive migrants, drugs will likely remain ineffective in endemic areas with similar epidemiologic characteristics such as the one studied here. Although several factors could affect the number of malaria cases, variation in the genetic composition of *Plasmodium* populations provides additional information that can facilitate the understanding of such changes and, by so doing, support science-based decision making process regarding malaria control policies.

## Additional files

**Additional file 1.** Fragment frequencies by year considering complete haplotype information per sample.
**Additional file 2.** Total haplotypes inferred from neutral microsatellite loci (persistent in gray).
**Additional file 3.** Unique haplotypes per year inferred from neutral microsatellite loci.
**Additional file 4.** *Pfcrt* linked microsatellite loci and mutation sites.
**Additional file 5.** *Pfdhfr* linked microsatellite loci and mutation sites.
**Additional file 6.** *Pfdhps* linked microsatellite loci and mutation sites.
