# Supplementary material for: Longitudinal analysis of Plasmodium falciparum genetic variation in Turbo, Colombia: implications for malaria control and elimination
Source: Malar J. 2015 Sep 22;14:363. doi: 10.1186/s12936-015-0887-9 (PMC4578328; doi:10.1186/s12936-015-0887-9)
Supplement: Supplementary file 1 — Additional file 1. Fragment frequencies by year considering complete haplotype information per sample. [file 12936_2015_887_MOESM1_ESM.docx]

**Additional file 1. Fragment frequencies by year considering complete haplotype information per sample.**

Figure 1A

Figure 1B

Figure 1C

Figure 1D

Figure 1E

Figure 1F

Figure 1G

Figure 1H
