# Supplementary material for: Longitudinal analysis of Plasmodium falciparum genetic variation in Turbo, Colombia: implications for malaria control and elimination
Source: Malar J. 2015 Sep 22;14:363. doi: 10.1186/s12936-015-0887-9 (PMC4578328; doi:10.1186/s12936-015-0887-9)
Supplement: Supplementary file 2 — Additional file 2. Total haplotypes inferred from neutral microsatellite loci (persistent in gray). [file 12936_2015_887_MOESM2_ESM.docx]

**Additional file 2.** Total haplotypes inferred from neutral microsatellite loci (persistent in gray).

| No. | Freq. | POLYa | TA60 | ARA2 | Pfg377 | PfPK2 | TA109 | TA81 | TA42 | 2490 |
| --- | --- | --- | --- | --- | --- | --- | --- | --- | --- | --- |
| haplo-1 | 0.008 | 146 | 79 | 69 | 95 | 171 | 162 | 127 | 185 | 81 |
| haplo-2 | 0.012 | 148 | 79 | 55 | 95 | 158 | 162 | 121 | 185 | 81 |
| haplo-3 | 0.012 | 148 | 79 | 55 | 95 | 162 | 162 | 121 | 185 | 81 |
| haplo-4 | 0.008 | 148 | 79 | 55 | 95 | 173 | 162 | 127 | 185 | 81 |
| haplo-5 | 0.023 | 148 | 79 | 65 | 95 | 158 | 162 | 127 | 185 | 81 |
| haplo-6 | 0.004 | 148 | 79 | 65 | 95 | 160 | 162 | 127 | 185 | 81 |
| haplo-7 | 0.004 | 148 | 79 | 65 | 95 | 162 | 162 | 115 | 185 | 81 |
| haplo-8 | 0.004 | 148 | 79 | 65 | 95 | 162 | 162 | 124 | 185 | 78 |
| haplo-9 | 0.004 | 148 | 79 | 65 | 95 | 162 | 162 | 127 | 185 | 74 |
| haplo-10 | 0.016 | 148 | 79 | 65 | 95 | 162 | 162 | 127 | 185 | 78 |
| haplo-11 | 0.141 | 148 | 79 | 65 | 95 | 162 | 162 | 127 | 185 | 81 |
| haplo-12 | 0.008 | 148 | 79 | 65 | 95 | 162 | 162 | 127 | 185 | 86 |
| haplo-13 | 0.004 | 148 | 79 | 65 | 95 | 162 | 177 | 127 | 185 | 86 |
| haplo-14 | 0.004 | 148 | 79 | 65 | 95 | 165 | 162 | 121 | 185 | 74 |
| haplo-15 | 0.020 | 148 | 79 | 65 | 95 | 168 | 162 | 121 | 185 | 74 |
| haplo-16 | 0.004 | 148 | 79 | 65 | 95 | 171 | 162 | 127 | 185 | 81 |
| haplo-17 | 0.004 | 148 | 79 | 65 | 95 | 173 | 162 | 121 | 182 | 81 |
| haplo-18 | 0.020 | 148 | 79 | 65 | 95 | 173 | 162 | 121 | 185 | 81 |
| haplo-19 | 0.004 | 148 | 79 | 65 | 95 | 173 | 162 | 127 | 182 | 81 |
| haplo-20 | 0.043 | 148 | 79 | 65 | 95 | 173 | 162 | 127 | 185 | 81 |
| haplo-21 | 0.004 | 148 | 79 | 65 | 95 | 173 | 162 | 127 | 185 | 84 |
| haplo-22 | 0.004 | 148 | 79 | 65 | 95 | 175 | 162 | 127 | 185 | 81 |
| haplo-23 | 0.004 | 148 | 79 | 69 | 95 | 162 | 162 | 121 | 185 | 81 |
| haplo-24 | 0.004 | 148 | 79 | 69 | 95 | 162 | 162 | 127 | 185 | 78 |
| haplo-25 | 0.004 | 148 | 79 | 69 | 95 | 162 | 162 | 127 | 185 | 86 |
| haplo-26 | 0.027 | 148 | 79 | 69 | 95 | 171 | 162 | 121 | 185 | 74 |
| haplo-27 | 0.008 | 148 | 79 | 69 | 95 | 171 | 162 | 121 | 185 | 81 |
| haplo-28 | 0.012 | 148 | 79 | 69 | 95 | 171 | 162 | 127 | 185 | 81 |
| haplo-29 | 0.047 | 148 | 79 | 69 | 95 | 173 | 162 | 121 | 185 | 74 |
| haplo-30 | 0.039 | 148 | 79 | 69 | 95 | 173 | 162 | 121 | 185 | 81 |
| haplo-31 | 0.004 | 148 | 79 | 69 | 95 | 173 | 162 | 121 | 185 | 86 |
| haplo-32 | 0.105 | 148 | 79 | 69 | 95 | 173 | 162 | 127 | 185 | 81 |
| haplo-33 | 0.004 | 148 | 79 | 69 | 95 | 173 | 162 | 127 | 185 | 86 |
| haplo-34 | 0.004 | 148 | 81 | 65 | 95 | 173 | 162 | 127 | 185 | 81 |
| haplo-35 | 0.004 | 151 | 79 | 55 | 95 | 162 | 162 | 121 | 185 | 81 |
| haplo-36 | 0.008 | 151 | 79 | 65 | 95 | 158 | 162 | 127 | 185 | 81 |
| haplo-37 | 0.008 | 151 | 79 | 65 | 95 | 162 | 162 | 127 | 185 | 81 |
| haplo-38 | 0.008 | 151 | 79 | 69 | 95 | 171 | 162 | 121 | 185 | 74 |
| haplo-39 | 0.008 | 151 | 79 | 69 | 95 | 171 | 162 | 121 | 185 | 81 |
| haplo-40 | 0.012 | 151 | 79 | 69 | 95 | 171 | 162 | 127 | 185 | 81 |

**Additional file 2 (continue).**

| No. | Freq. | POLYa | TA60 | ARA2 | Pfg377 | PfPK2 | TA109 | TA81 | TA42 | 2490 |
| --- | --- | --- | --- | --- | --- | --- | --- | --- | --- | --- |
| haplo-41 | 0.004 | 151 | 79 | 69 | 95 | 173 | 162 | 121 | 185 | 74 |
| haplo-42 | 0.004 | 151 | 79 | 69 | 95 | 173 | 162 | 121 | 185 | 81 |
| haplo-43 | 0.004 | 155 | 79 | 57 | 95 | 171 | 162 | 127 | 185 | 74 |
| haplo-44 | 0.004 | 158 | 79 | 65 | 95 | 171 | 162 | 121 | 185 | 74 |
| haplo-45 | 0.004 | 158 | 79 | 65 | 95 | 171 | 162 | 121 | 185 | 86 |
| haplo-46 | 0.023 | 158 | 79 | 65 | 95 | 173 | 162 | 121 | 185 | 74 |
| haplo-47 | 0.004 | 158 | 79 | 65 | 95 | 173 | 162 | 121 | 185 | 78 |
| haplo-48 | 0.004 | 158 | 79 | 65 | 95 | 173 | 162 | 121 | 185 | 81 |
| haplo-49 | 0.004 | 158 | 79 | 65 | 95 | 173 | 162 | 127 | 185 | 74 |
| haplo-50 | 0.004 | 158 | 79 | 65 | 95 | 173 | 162 | 127 | 185 | 78 |
| haplo-51 | 0.008 | 158 | 79 | 65 | 95 | 173 | 162 | 127 | 185 | 81 |
| haplo-52 | 0.004 | 158 | 79 | 69 | 95 | 162 | 162 | 121 | 185 | 74 |
| haplo-53 | 0.004 | 158 | 79 | 69 | 95 | 162 | 162 | 127 | 182 | 81 |
| haplo-54 | 0.004 | 158 | 79 | 69 | 95 | 162 | 162 | 127 | 185 | 74 |
| haplo-55 | 0.016 | 158 | 79 | 69 | 95 | 162 | 162 | 127 | 185 | 81 |
| haplo-56 | 0.004 | 162 | 79 | 55 | 95 | 162 | 162 | 121 | 185 | 74 |
| haplo-57 | 0.004 | 162 | 79 | 55 | 95 | 162 | 162 | 121 | 185 | 78 |
| haplo-58 | 0.027 | 162 | 79 | 55 | 95 | 162 | 162 | 121 | 185 | 81 |
| haplo-59 | 0.008 | 162 | 79 | 55 | 95 | 162 | 162 | 121 | 185 | 86 |
| haplo-60 | 0.012 | 162 | 79 | 55 | 95 | 173 | 162 | 121 | 185 | 74 |
| haplo-61 | 0.004 | 162 | 79 | 55 | 95 | 173 | 162 | 121 | 185 | 86 |
| haplo-62 | 0.004 | 162 | 79 | 55 | 95 | 173 | 162 | 127 | 185 | 81 |
| haplo-63 | 0.004 | 162 | 79 | 55 | 95 | 173 | 162 | 127 | 185 | 86 |
| haplo-64 | 0.004 | 162 | 79 | 65 | 95 | 158 | 162 | 127 | 185 | 74 |
| haplo-65 | 0.004 | 162 | 79 | 65 | 95 | 162 | 162 | 121 | 185 | 74 |
| haplo-66 | 0.008 | 162 | 79 | 65 | 95 | 162 | 162 | 121 | 185 | 81 |
| haplo-67 | 0.004 | 162 | 79 | 65 | 95 | 162 | 162 | 127 | 185 | 81 |
| haplo-68 | 0.008 | 162 | 79 | 65 | 95 | 171 | 162 | 121 | 185 | 74 |
| haplo-69 | 0.016 | 162 | 79 | 65 | 95 | 171 | 162 | 127 | 185 | 74 |
| haplo-70 | 0.004 | 162 | 79 | 65 | 95 | 173 | 162 | 121 | 185 | 74 |
| haplo-71 | 0.004 | 162 | 79 | 65 | 95 | 173 | 162 | 127 | 182 | 74 |
| haplo-72 | 0.063 | 162 | 79 | 65 | 95 | 173 | 162 | 127 | 185 | 74 |
| haplo-73 | 0.012 | 162 | 79 | 65 | 95 | 173 | 162 | 127 | 185 | 81 |
| haplo-74 | 0.020 | 162 | 79 | 69 | 95 | 162 | 162 | 121 | 185 | 81 |
| haplo-75 | 0.004 | 162 | 79 | 69 | 95 | 171 | 162 | 127 | 185 | 81 |
| haplo-76 | 0.004 | 162 | 79 | 69 | 95 | 173 | 162 | 127 | 185 | 78 |
| haplo-77 | 0.023 | 162 | 79 | 69 | 95 | 173 | 162 | 127 | 185 | 81 |
| haplo-78 | 0.004 | 162 | 79 | 69 | 95 | 173 | 162 | 127 | 185 | 86 |
| haplo-79* | 0.000 | 181 | 79 | 65 | 102 | 191 | 189 | 115 | 185 | 84 |

*Migrant haplotype. The haplotypes highlighted are those found across the 6 years of the study.
