# Supplementary material for: Longitudinal analysis of Plasmodium falciparum genetic variation in Turbo, Colombia: implications for malaria control and elimination
Source: Malar J. 2015 Sep 22;14:363. doi: 10.1186/s12936-015-0887-9 (PMC4578328; doi:10.1186/s12936-015-0887-9)
Supplement: Supplementary file 3 — Additional file 3. Unique haplotypes per year inferred from neutral microsatellite loci. [file 12936_2015_887_MOESM3_ESM.docx]

**Additional file 3.** Unique haplotypes per year inferred from neutral microsatellite loci.

| Haplotype Code | Year |  | Haplotype Code | Year |
| --- | --- | --- | --- | --- |
| haplo-15 | 2002 |  | haplo-18 | 2004 |
| haplo-16 | 2002 |  | haplo-22 | 2004 |
| haplo-30 | 2002 |  | haplo-24 | 2004 |
| haplo-31 | 2002 |  | haplo-25 | 2004 |
| haplo-34 | 2002 |  | haplo-27 | 2004 |
| haplo-43 | 2002 |  | haplo-28 | 2004 |
| haplo-45 | 2002 |  | haplo-46 | 2004 |
| haplo-54 | 2002 |  | haplo-50 | 2004 |
| haplo-55 | 2002 |  | haplo-53 | 2004 |
| haplo-6 | 2003 |  | haplo-3 | 2005 |
| haplo-7 | 2003 |  | haplo-9 | 2005 |
| haplo-14 | 2003 |  | haplo-13 | 2005 |
| haplo-19 | 2003 |  | haplo-17 | 2005 |
| haplo-20 | 2003 |  | haplo-23 | 2005 |
| haplo-29 | 2003 |  | haplo-26 | 2005 |
| haplo-32 | 2003 |  | haplo-4 | 2007 |
| haplo-33 | 2003 |  | haplo-5 | 2007 |
| haplo-37 | 2003 |  | haplo-8 | 2007 |
| haplo-39 | 2003 |  | haplo-11 | 2007 |
| haplo-40 | 2003 |  | haplo-12 | 2007 |
| haplo-41 | 2003 |  | haplo-21 | 2007 |
| haplo-42 | 2003 |  | haplo-35 | 2007 |
| haplo-44 | 2003 |  | haplo-52 | 2007 |
| haplo-48 | 2003 |  | haplo-10 | 2008 |
| haplo-49 | 2003 |  | haplo-36 | 2008 |
| haplo-51 | 2003 |  | haplo-38 | 2008 |
| haplo-1 | 2004 |  | haplo-47 | 2008 |
| haplo-2 | 2004 |  |  |  |
