# Supplementary material for: Longitudinal analysis of Plasmodium falciparum genetic variation in Turbo, Colombia: implications for malaria control and elimination
Source: Malar J. 2015 Sep 22;14:363. doi: 10.1186/s12936-015-0887-9 (PMC4578328; doi:10.1186/s12936-015-0887-9)
Supplement: Supplementary file 4 — Additional file 4. Pfcrt linked microsatellite loci and mutation sites. [file 12936_2015_887_MOESM4_ESM.docx]

**Additional file 4.** *Pfcrt* linked microsatellite loci and mutation sites.

| No. | Freq. | -257 | -200 | -45 | -4.8 | -4.5 | 4.6 | 7 | 48 | 60 | 245 | 72 | 73 | 74 | 75 | 76 |
| --- | --- | --- | --- | --- | --- | --- | --- | --- | --- | --- | --- | --- | --- | --- | --- | --- |
| 1 | 0.005 | 195 | 180 | 114 | 183 | 233 | 157 | 307 | 114 | 126 | 191 | C | V | M | E | T |
| 2 | 0.01 | 195 | 182 | 111 | 183 | 228 | 157 | 307 | 114 | 126 | 191 | C | V | M | E | T |
| 3 | 0.005 | 195 | 180 | 114 | 183 | 228 | 157 | 307 | 114 | 123 | 191 | C | V | M | E | T |
| 4 | 0.005 | 195 | 180 | 114 | 183 | 228 | 157 | 307 | 114 | 123 | 181 | C | V | M | E | T |
| 5 | 0.168 | 195 | 180 | 114 | 183 | 228 | 157 | 307 | 114 | 140 | 181 | C | V | M | E | T |
| 6 | 0.021 | 181 | 177 | 114 | 183 | 228 | 157 | 307 | 114 | 140 | 191 | C | V | M | E | T |
| 7 | 0.225 | 195 | 177 | 114 | 183 | 228 | 157 | 307 | 114 | 126 | 191 | C | V | M | E | T |
| 8 | 0.016 | 195 | 177 | 114 | 183 | 228 | 157 | 307 | 114 | 126 | 181 | C | V | M | E | T |
| 9 | 0.089 | 195 | 177 | 114 | 185 | 228 | 157 | 307 | 114 | 126 | 191 | C | V | M | E | T |
| 10 | 0.304 | 195 | 180 | 114 | 183 | 228 | 157 | 307 | 114 | 126 | 191 | C | V | I | E | T |
| 11 | 0.005 | 179 | 177 | 114 | 183 | 228 | 157 | 307 | 114 | 140 | 191 | C | V | M | E | T |
| 12 | 0.016 | 195 | 177 | 114 | 185 | 230 | 157 | 307 | 114 | 126 | 191 | C | V | M | E | T |
| 13 | 0.01 | 195 | 182 | 114 | 183 | 228 | 157 | 307 | 114 | 126 | 191 | C | V | M | E | T |
| 14 | 0.01 | 179 | 177 | 114 | 183 | 228 | 157 | 307 | 114 | 126 | 181 | C | V | M | E | T |
| 15 | 0.005 | 195 | 177 | 114 | 183 | 228 | 157 | 307 | 114 | 140 | 191 | C | V | M | E | T |
| 16 | 0.005 | 195 | 177 | 114 | 183 | 228 | 157 | 307 | 114 | 126 | 192 | C | V | M | E | T |
| 17 | 0.01 | 195 | 180 | 114 | 183 | 230 | 157 | 307 | 114 | 140 | 181 | C | V | M | E | T |
| 18 | 0.01 | 195 | 180 | 114 | 183 | 228 | 157 | 307 | 114 | 126 | 181 | C | V | M | E | T |
| 19 | 0.005 | 181 | 180 | 114 | 183 | 228 | 157 | 307 | 114 | 140 | 191 | C | V | M | E | T |
| 20 | 0.005 | 196 | 180 | 114 | 183 | 228 | 157 | 307 | 114 | 126 | 181 | C | V | M | E | T |
| 21 | 0.016 | 195 | 180 | 114 | 183 | 230 | 157 | 307 | 114 | 126 | 191 | C | V | M | E | T |
| 22 | 0.021 | 195 | 180 | 114 | 183 | 228 | 157 | 307 | 114 | 147 | 181 | C | V | M | E | T |
| 23 | 0.005 | 195 | 180 | 114 | 183 | 228 | 157 | 307 | 114 | 137 | 182 | C | V | M | E | T |
| 24 | 0.005 | 195 | 180 | 111 | 183 | 228 | 157 | 307 | 114 | 140 | 181 | C | V | M | E | T |
| 25 | 0.005 | 181 | 177 | 114 | 183 | 228 | 157 | 307 | 114 | 126 | 181 | C | V | M | E | T |
| 26 | 0.005 | 189 | 177 | 114 | 183 | 228 | 157 | 307 | 114 | 126 | 191 | C | V | M | E | T |
| 27 | 0.005 | 189 | 177 | 114 | 183 | 228 | 160 | 307 | 114 | 126 | 187 | C | V | M | E | T |
| 28 | 0.005 | 189 | 177 | 114 | 185 | 228 | 157 | 307 | 114 | 126 | 181 | C | V | M | E | T |
| * |  | 179 | 180 | ? | 166 | 233 | 167 | 307 | 126 | 147 | 181 | C | V | M | N | K |

*Migrant haplotype.
