# Supplementary material for: Longitudinal analysis of Plasmodium falciparum genetic variation in Turbo, Colombia: implications for malaria control and elimination
Source: Malar J. 2015 Sep 22;14:363. doi: 10.1186/s12936-015-0887-9 (PMC4578328; doi:10.1186/s12936-015-0887-9)
Supplement: Supplementary file 5 — Additional file 5. Pfdhfr linked microsatellite loci and mutation sites. [file 12936_2015_887_MOESM5_ESM.docx]

**Additional file 5.** *Pfdhfr* linked microsatellite loci and mutation sites.

| No. | Freq. | -89 | -58 | -7.058 | -4.973 | -4.423 | -4.12 | -3.77 | -1.14 | -0.064 | 0.105 | 0.45 | 1.33 | 5.782 | 18.209 | 39.91 | 50 | 51 | 59 | 108 | 164 |
| --- | --- | --- | --- | --- | --- | --- | --- | --- | --- | --- | --- | --- | --- | --- | --- | --- | --- | --- | --- | --- | --- |
| 1 | 0.50323 | 139 | 161 | 258 | 200 | 197 | 175 | 216 | 223 | 96 | 159 | 100 | 203 | 107 | 282 | 185 | C | **I** | C | **N** | I |
| 2 | 0.00645 | 139 | 161 | 258 | 200 | 197 | 175 | 216 | 223 | 96 | 162 | 100 | 203 | 107 | 282 | 185 | C | **I** | C | **N** | I |
| 3 | 0.00645 | 137 | 161 | 258 | 200 | 197 | 175 | 216 | 232 | 96 | 159 | 100 | 203 | 107 | 282 | 185 | C | **I** | C | **N** | I |
| 4 | 0.07742 | 139 | 161 | 258 | 200 | 197 | 175 | 212 | 223 | 96 | 159 | 100 | 203 | 107 | 282 | 188 | C | **I** | C | **N** | I |
| 5 | 0.05161 | 139 | 161 | 258 | 200 | 197 | 175 | 214 | 223 | 96 | 159 | 100 | 203 | 107 | 282 | 185 | C | **I** | C | **N** | I |
| 6 | 0.01936 | 139 | 161 | 258 | 200 | 200 | 175 | 212 | 223 | 96 | 159 | 100 | 203 | 107 | 282 | 188 | C | **I** | C | **N** | I |
| 7 | 0.05161 | 139 | 161 | 258 | 200 | 200 | 175 | 216 | 223 | 96 | 159 | 100 | 203 | 107 | 282 | 185 | C | **I** | C | **N** | I |
| 8 | 0.0129 | 124 | 161 | 258 | 200 | 197 | 175 | 216 | 223 | 96 | 159 | 100 | 203 | 107 | 282 | 185 | C | **I** | C | **N** | I |
| 9 | 0.01936 | 124 | 161 | 258 | 200 | 197 | 175 | 214 | 223 | 96 | 159 | 100 | 203 | 107 | 282 | 185 | C | **I** | C | **N** | I |
| 10 | 0.02581 | 139 | 161 | 258 | 200 | 197 | 175 | 214 | 223 | 96 | 159 | 100 | 203 | 107 | 282 | 188 | C | **I** | C | **N** | I |
| 11 | 0.00645 | 139 | 161 | 258 | 200 | 200 | 175 | 212 | 223 | 94 | 159 | 100 | 203 | 107 | 282 | 188 | C | **I** | C | **N** | I |
| 12 | 0.00645 | 139 | 161 | 258 | 200 | 200 | 175 | 216 | 223 | 96 | 159 | 100 | 203 | 107 | 282 | 188 | C | **I** | C | **N** | I |
| 13 | 0.10968 | 139 | 161 | 258 | 200 | 197 | 175 | 210 | 223 | 96 | 159 | 100 | 203 | 107 | 282 | 185 | C | **I** | C | **N** | I |
| 14 | 0.00645 | 139 | 161 | 258 | 200 | 197 | 175 | 216 | 232 | 96 | 159 | 100 | 203 | 107 | 282 | 185 | C | **I** | C | **N** | I |
| 15 | 0.00645 | 137 | 161 | 258 | 204 | 197 | 175 | 216 | 223 | 96 | 159 | 100 | 203 | 107 | 282 | 185 | C | **I** | C | **N** | I |
| 16 | 0.00645 | 139 | 161 | 258 | 200 | 197 | 175 | 216 | 223 | 94 | 159 | 100 | 203 | 107 | 282 | 188 | C | **I** | C | **N** | I |
| 17 | 0.00645 | 139 | 161 | 258 | 200 | 197 | 175 | 212 | 223 | 94 | 159 | 100 | 203 | 107 | 282 | 185 | C | **I** | C | **N** | I |
| 18 | 0.00645 | 139 | 161 | 258 | 200 | 197 | 175 | 210 | 223 | 94 | 159 | 100 | 203 | 107 | 282 | 185 | C | **I** | C | **N** | I |
| 19 | 0.00645 | 137 | 158 | 258 | 200 | 197 | 175 | 216 | 223 | 96 | 159 | 100 | 203 | 107 | 282 | 185 | C | **I** | C | **N** | I |
| 20 | 0.00645 | 139 | 161 | 258 | 200 | 197 | 175 | 208 | 223 | 96 | 159 | 100 | 203 | 107 | 282 | 183 | C | **I** | C | **N** | I |
| 21 | 0.0129 | 137 | 161 | 258 | 200 | 197 | 175 | 216 | 223 | 96 | 159 | 100 | 203 | 107 | 282 | 185 | C | **I** | C | **N** | I |
| 22 | 0.00645 | 139 | 161 | 258 | 200 | 197 | 175 | 216 | 200 | 96 | 159 | 100 | 203 | 107 | 282 | 185 | C | **I** | C | **N** | I |
| 23 | 0.0129 | 139 | 161 | 258 | 200 | 197 | 175 | 208 | 223 | 96 | 159 | 100 | 203 | 107 | 282 | 185 | C | **I** | C | **N** | I |
| 24 | 0.00645 | 137 | 161 | 258 | 200 | 197 | 175 | 214 | 223 | 96 | 159 | 100 | 203 | 107 | 282 | 185 | C | **I** | C | **N** | I |
| 25 | 0.00645 | 139 | 163 | 258 | 200 | 197 | 175 | 208 | 223 | 96 | 159 | 100 | 203 | 107 | 282 | 185 | C | **I** | C | **N** | I |
| 26 | 0.00645 | 139 | 163 | 258 | 200 | 197 | 179 | 216 | 223 | 96 | 159 | 100 | 203 | 107 | 282 | 195 | C | **I** | C | **N** | I |
| 27 | 0.00645 | 139 | 161 | 258 | 200 | 197 | 175 | 186 | 206 | 96 | 159 | 100 | 203 | 107 | 282 | 185 | C | **I** | C | **N** | I |
| * |  | ? | ? | 260 | 222 | 201 | 186 | 212 | 211 | 100 | 177 | 81 | 203 | 111 | 282 | 173 | C | **N** | C | **S** | I |

*Migrant haplotype.
