# Supplementary material for: Longitudinal analysis of Plasmodium falciparum genetic variation in Turbo, Colombia: implications for malaria control and elimination
Source: Malar J. 2015 Sep 22;14:363. doi: 10.1186/s12936-015-0887-9 (PMC4578328; doi:10.1186/s12936-015-0887-9)
Supplement: Supplementary file 6 — Additional file 6. Pfdhps linked microsatellite loci and mutation sites. [file 12936_2015_887_MOESM6_ESM.docx]

**Additional file 6.** *Pfdhps* linked microsatellite loci and mutation sites.

| No. | Freq | -72.8 | -34.6 | -18.8 | -11.1 | -7.5 | -2.8 | -1.5 | -0.1 | 0.5 | 1.4 | 3.4 | 6.4 | 9.0 | 49.6 | 66.1 | 436 | 437 | 540 | 581 | 613 |
| --- | --- | --- | --- | --- | --- | --- | --- | --- | --- | --- | --- | --- | --- | --- | --- | --- | --- | --- | --- | --- | --- |
| 1 | 0.310 | 178 | 206 | 101 | 219 | 169 | 181 | 168 | 132 | 140 | 238 | 108 | 291 | 105 | 300 | 329 | S | **G** | K | A | A |
| 2 | 0.297 | 178 | 206 | 101 | 219 | 169 | 181 | 168 | 132 | 140 | 238 | 108 | 291 | 105 | 296 | 329 | S | **G** | K | A | A |
| 3 | 0.007 | 178 | 206 | 95 | 219 | 173 | 181 | 165 | 132 | 140 | 265 | 108 | 287 | 107 | 283 | 329 | S | **A** | K | A | A |
| 4 | 0.179 | 178 | 206 | 101 | 219 | 169 | 181 | 168 | 132 | 140 | 238 | 108 | 301 | 105 | 300 | 329 | S | **G** | K | A | A |
| 5 | 0.007 | 178 | 206 | 101 | 219 | 169 | 181 | 168 | 132 | 140 | 238 | 108 | 291 | 105 | 293 | 329 | S | **G** | K | A | A |
| 6 | 0.007 | 178 | 206 | 101 | 219 | **167** | 181 | 168 | 132 | 140 | 238 | 108 | 291 | 105 | 296 | 329 | S | **G** | K | A | A |
| 7 | 0.007 | 178 | 206 | 95 | 219 | 169 | 181 | 165 | 134 | 140 | 265 | 106 | 287 | 107 | 296 | 329 | S | **A** | K | A | A |
| 8 | 0.007 | 178 | 206 | 101 | 219 | 169 | 181 | 168 | 134 | 140 | 238 | 106 | 291 | 105 | 300 | 329 | S | **G** | K | A | A |
| 9 | 0.007 | 178 | 206 | 101 | 219 | 169 | 181 | 168 | 132 | 140 | 238 | 108 | 291 | 105 | 283 | 329 | S | **G** | K | A | A |
| 10 | 0.007 | 178 | 206 | 101 | 219 | 173 | 181 | 168 | 132 | 140 | 238 | 108 | 301 | 105 | 300 | 329 | S | **G** | K | A | A |
| 11 | 0.007 | 178 | 206 | 101 | 219 | 169 | 181 | 168 | 134 | 140 | 238 | 108 | 291 | 105 | 296 | 329 | S | **G** | K | A | A |
| 12 | 0.007 | 178 | 206 | 101 | 228 | 177 | 197 | 165 | 132 | 140 | 269 | 108 | 291 | 133 | 283 | 329 | S | **A** | K | A | A |
| 13 | 0.007 | 178 | 206 | 95 | 219 | 169 | 181 | 165 | 132 | 140 | 265 | 106 | 287 | 107 | 283 | 329 | S | **A** | K | A | A |
| 14 | 0.007 | 178 | 206 | 95 | 219 | 169 | 181 | 165 | 132 | 140 | 265 | 108 | 287 | 107 | 283 | 329 | S | **A** | K | A | A |
| 15 | 0.014 | 178 | 206 | 101 | 219 | 169 | 181 | 168 | 132 | 136 | 238 | 108 | 291 | 105 | 300 | 329 | S | **G** | K | A | A |
| 16 | 0.007 | 178 | 206 | 101 | 219 | 169 | 181 | 168 | 132 | 136 | 238 | 108 | 291 | 105 | 296 | 329 | S | **G** | K | A | A |
| 17 | 0.034 | 178 | 206 | 101 | 219 | 169 | 181 | 168 | 132 | 140 | 238 | 108 | 301 | 105 | 296 | 329 | S | **G** | K | A | A |
| 18 | 0.007 | 178 | 206 | 95 | 219 | 169 | 181 | 165 | 134 | 140 | 265 | 106 | 287 | 107 | 283 | 329 | S | **A** | K | A | A |
| 19 | 0.007 | 180 | 206 | 101 | 228 | 177 | 197 | 168 | 134 | 140 | 269 | 108 | 291 | 133 | 283 | 329 | S | **A** | K | A | A |
| 20 | 0.007 | 178 | 206 | 95 | 219 | 169 | 181 | 165 | 134 | 140 | 265 | 104 | 287 | 107 | 283 | 329 | S | **A** | K | A | A |
| 21 | 0.007 | 178 | 206 | 101 | 219 | 169 | 181 | 168 | 132 | 140 | 238 | 111 | 291 | 105 | 300 | 329 | S | **G** | K | A | A |
| 22 | 0.007 | 180 | 206 | 101 | 228 | 177 | 197 | 168 | 134 | 140 | 275 | 108 | 291 | 105 | 283 | 329 | S | **A** | K | A | A |
| 23 | 0.007 | 178 | 206 | 101 | 219 | 173 | 181 | 168 | 132 | 140 | 238 | 108 | 291 | 105 | 300 | 329 | S | **G** | K | A | A |
| 24 | 0.007 | 178 | 206 | 95 | 219 | 169 | 181 | 168 | 132 | 140 | 238 | 108 | 291 | 105 | 296 | 329 | S | **G** | K | A | A |
| 25 | 0.007 | 180 | 206 | 95 | 219 | 169 | 181 | 168 | 132 | 140 | 238 | 108 | 291 | 105 | 283 | 329 | S | **A** | K | A | A |
| 26 | 0.007 | 180 | 206 | 101 | 228 | 177 | 197 | 165 | 134 | 140 | 269 | 108 | 291 | 133 | 283 | 329 | S | **A** | K | A | A |
| 27 | 0.007 | 180 | 206 | 95 | 219 | 169 | 181 | 168 | 132 | 140 | 238 | 108 | 291 | 105 | 296 | 329 | S | **A** | K | A | A |
| 28 | 0.007 | 180 | 206 | 101 | 228 | 177 | 197 | 165 | 134 | 140 | 262 | 108 | 291 | 133 | 283 | 329 | S | **A** | K | A | A |
| ***** | 0.007 | 204 | 208 | 88 | 219 | 169 | 197 | 165 | 134 | 140 | 248 | 102 | 287 | 101 | 287 | 322 | S | **A** | K | A | A |

*Migrant haplotype.
